# Recent advances in understanding mesenchymal stromal cells

**DOI:** 10.12688/f1000research.21862.1

**Published:** 2020-02-27

**Authors:** Erika Rendra, Eleonora Scaccia, Karen Bieback

**Affiliations:** 1 Institute of Transfusion Medicine and Immunology, Mannheim Institute of Innate Immunoscience, Medical Faculty Mannheim, Heidelberg University, Mannheim, 68167, Germany; 2 FlowCore Mannheim, Medical Faculty Mannheim, Heidelberg University, Mannheim, 68167, Germany; 3 German Red Cross Blood Donor Service Baden-Württemberg – Hessen, Mannheim, 68167, Germany

**Keywords:** mesenchymal stromal cells, translation, clinical perspectives, secretome, extracellular vesicles

## Abstract

Mesenchymal stromal cells (MSCs) are among of the most studied cell type for cellular therapy thanks to the ease of isolation, cultivation, and the high *ex vivo* expansion potential. In 2018, the European Medicines Agency finally granted the first marketing authorization for an MSC product. Despite the numerous promising results in preclinical studies, translation into routine practice still lags behind: therapeutic benefits of MSCs are not as satisfactory in clinical trial settings as they appear to be in preclinical models. The bench-to-bedside-and-back approach and careful evaluation of discrepancies between preclinical and clinical results have provided valuable insights into critical components of MSC manufacturing, their mechanisms of action, and how to evaluate and quality-control them. We sum up these past developments in the introductory section (“Mesenchymal stromal cells: name follows function”). From the huge amount of information, we then selected a few examples to illustrate challenges and opportunities to improve MSCs for clinical purposes. These include tissue origin of MSCs, MSC culture conditions, immune compatibility, and route of application and dosing. Finally, we add some information on MSC mechanisms of action and translation into potency assays and give an outlook on future perspectives raising the question of whether the future clinical product may be cell-based or cell-derived.

## Mesenchymal stromal cells: name follows function

In 2018, the first marketing authorization for a mesenchymal stromal cell (MSC) product was granted by the European Medicines Agency for the treatment of complex perianal fistulas in patients with Crohn’s disease ^1^. From a regulatory perspective, MSCs are classified as an advanced therapy medicinal product (ATMP) ( https://www.ema.europa.eu/en/human-regulatory/overview/advanced-therapy-medicinal-products-overview). This represents a milestone in the long history of MSCs, which were first described in 1867 by Cohnheim as non-hematopoietic bone marrow–derived cells to migrate through the bloodstream to distant sites of injury and participate in tissue regeneration ^2^. In the 1970s, Friedenstein *et al*. characterized them as a minor subpopulation of marrow-derived plastic adherent cells with osteogenic and hematopoietic supportive potential ^3^. He also established the term colony-forming unit-fibroblast. In the 1990s, Caplan ^4, 5^ and Pittenger *et al*. ^6^ coined the term “mesenchymal stem cells” on the basis of the multi-lineage differentiation potential of these cells. At this time, controversy arose as to whether these cells are stem cells or not. Bianco and Robey and colleagues used the term “skeletal stem cells” for cells residing in the postnatal bone marrow and giving rise to cartilage, bone, hematopoiesis-supportive stroma, and marrow adipocytes in defined *in vivo* assays ^7– 9^. In 2006, to put an end to the discussion, the International Society for Cell and Gene Therapy defined the term “mesenchymal stromal cells” and set up minimal criteria defining (bone marrow–derived) MSCs ( Table 1) ^10^. At that time, it became evident that MSCs (or at least cells with similar characteristics) could be isolated from a variety of different tissues, suggesting a perivascular origin ^11, 12^. Given the similarity to fibroblasts, Haniffa *et al*. asked: “Mesenchymal stem cells: The fibroblasts’ new clothes?” ^13^. However, with the increasing use in (pre)clinical studies, it became evident that apparently not the proposed multi-lineage differentiation potential but rather their secreted bioactive molecules that modulate immune and inflammatory responses were key to exerting therapeutic effects (in fact, only few transplanted cells were found *in vivo* to be engrafted and differentiated) ^14, 15^. Thus, Caplan introduced the term “medicinal signaling cells” to illustrate their versatility and flexibility to adapt to the local milieu ^16^. We interpret the abbreviation “MSCs” as “mesenchymal stromal cells” as, according to our own experimental observations, the cells do not fulfil “stem cell” criteria such as indefinite self-renewal.

**Table 1.** Definition of mesenchymal stromal cell.

Adherence to plastic	Specific surface markers	*In vitro* multipotent differentiation potential
	Positive: CD105 CD73 CD90 Negative: CD45 CD34 CD14 CD11b CD79a CD19 HLA class I	Osteoblasts Adipocytes Chondroblasts

The minimal criteria defining the mesenchymal stromal cell by the International Society for Cell and Gene Therapy (according to 10).

Despite arguments about the most appropriate name, MSCs have emerged as the most intensely studied cell type for experimental cell therapy. Starting with the first use in patients in a hematological transplant setting ^17^, numerous clinical indications have been investigated, ranging from hematological disorders (including graft-versus-host disease, or GvHD), bone/cartilage defects, diabetes, cardiovascular and neurological diseases (including autoimmune diseases), and liver and renal diseases ^18– 20^. The ease of isolation, cultivation, and the high *ex vivo* expansion potential in line with the numerous therapeutic mechanisms (paracrine pro-regenerative, anti-fibrotic, anti-apoptotic, pro-angiogenic, and immunomodulatory functions) have contributed to this broad exploitation.

## Challenges and opportunities to improve mesenchymal stromal cells for clinical purposes

**Figure 1.**
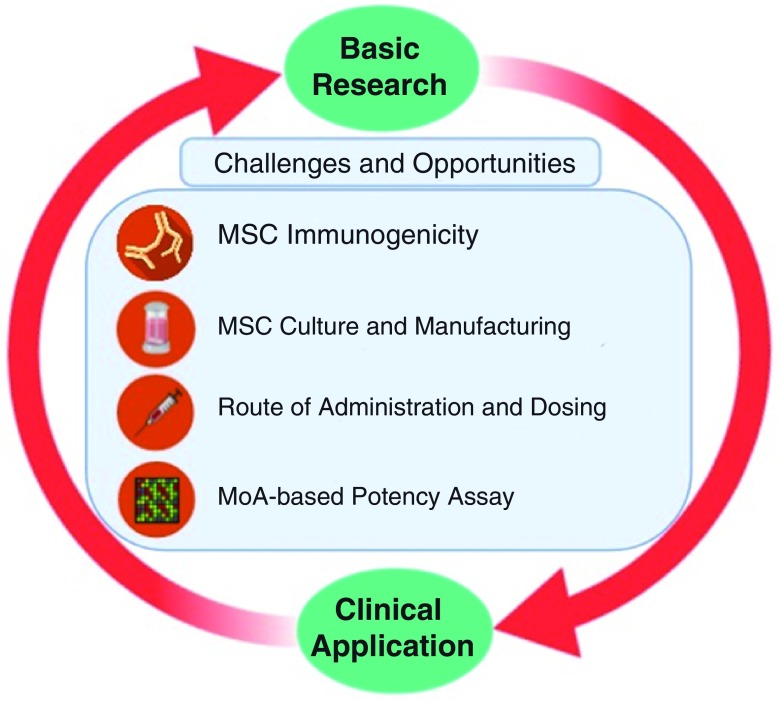
Bench-to-bedside-and-back. Challenges and opportunities in translating mesenchymal stromal cell (MSC)-based therapy from basic research to clinical practices, including immunogenicity of MSCs, Good Manufacturing Practice–compliant MSC manufacturing as well as determining the route of administration and dosing. MoA, mechanism of action.

Despite the promising results in preclinical studies, therapeutic benefits of MSCs are not as satisfactory in clinical trial settings ^21^. This section addresses some factors that might contribute to this disparity and how to improve the therapeutic capacity of MSCs ( Figure 1).

### Tissue origin of mesenchymal stromal cells

The most prevalent source for MSCs is adult bone marrow ^18^. Adipose tissue is emerging as an important source, as exemplified by the ATMP granted marketing authorization by the European Medicines Agency (mentioned above). We and others have tried to understand how interchangeable MSCs from different tissue sources are and whether one may be more suitable for certain disease entities than for others. The observed differences suggest an “environmental niche memory”, which could help to select the most appropriate tissue source for a certain clinical indication ^12, 22– 26^.

### Mesenchymal stromal cell culture conditions and cellular fitness

Usage of fetal bovine serum (FBS) as culture supplement has been a major issue in MSC production ^27^. The growing concern relates to transmission of pathogens such as prions and possible immune reactions against xenogeneic agents ^28, 29^. Consequently, as a replacement of FBS, other supplements have been introduced. One of the most common is platelet lysate (PL) ^29– 31^, as it contains growth factors suitable to support MSC *ex vivo* expansion without causing genomic instability ^32^. However, the use of PL is not without concerns: batch-to-batch variation and pathogen reduction need to be addressed to standardize PL use in MSC manufacturing ^33^.

MSC culture conditions differ enormously, hampering comparability of data ^34^. Cellular “fitness” is considered the most critical parameter and is influenced by cellular/replicative age and potential “cryo-injury” ^21, 35, 36^.

Expansion of MSCs *in vitro*, required to achieve clinical doses (see below), ultimately results in replicative senescence that compromises therapeutic efficacy ^37, 38^. Thus, genomic stability should be addressed as a safety measure before clinical application ^39^. In addition, the thawing of cryopreserved MSCs just before transplantation may hamper their therapeutic capacity. In most animal experiments, MSCs are harvested freshly before the transplantation, while on the peak of the replicative phase. Meanwhile, in clinical trial, most MSCs are pre-banked and expanded to their proliferative limit, frozen down, and just thawed prior the transplantation ^35, 40^. Following retrieval from liquid nitrogen, MSCs have been shown to undergo a heat shock response (“cryo stun effect”) leading to cell injury for at least the first 24 hours ^40^. This has been shown to compromise immune-modulation function, enhance vulnerability to lysis by immune cells and the complement system, and decrease *in vivo* persistence upon intravenous administration ^40^. A rescue culture for a few days could eventually reduce this “cryo stun effect”.

As it has become clear that culture conditions can greatly affect MSC function, it also opens a new window for MSC priming to improve their therapeutic efficacy. A growing body of data report a wide array of priming approaches, from usage of cytokines, growth factors, hypoxia, pharmaceutical drugs, and 3D culture using biomaterials ^41, 42^. For example, MSC priming with interferon-gamma (IFN-γ) is considered key to suppress T-cell proliferation, partly through production of indolamine-2,3-dioxygenase (IDO) and programmed cell death-1 ligand (PDL-1) upregulation ^43^. Indeed, allogeneic infusion of IFN-γ–primed MSCs to non-obese diabetic/severe combined immunodeficiency (NOD/SCID) mice reduced GvHD symptoms ^44^. However, MSC priming with IFN-γ should be carried out with caution as it can upregulate the expression of HLA class I and II molecules, which could affect immune compatibility ^45^.

Lastly, another challenge to bring MSCs to clinical application is upscaling of MSC culture. A number of strategies for upscaling cell, secretome, or extracellular vesicle production have been reported and reviewed extensively ^46– 48^. However, economically feasible approaches that meet Good Manufacturing Practice compliance have yet to be standardized ^49^.

### Route of application and dosing

Depending on the clinical purposes, MSCs are administered differently, either systemically infused or locally injected. Contrary to the old belief that MSCs migrate to the site of injury and replace the injured tissue once MSCs are injected intravenously, they are mostly trapped in lungs and die within 24 hours ^50^. Pulmonary embolism and infarct of three related patients have been reported after adipose MSC infusion ^51^. MSCs express tissue factor, a cell surface glycoprotein that plays an important role in extrinsic coagulation, which by triggering procoagulation has led to thromboembolic events after MSC infusion. Thus, adding an anti-coagulant during the infusion should be considered ^52^.

The majority of preclinical studies using mice and rats infuse around 50 million and 10 to 20 million MSCs per kilogram of body weight, respectively ^53, 54^. Meanwhile, the average number of MSCs transfused intravenously is 100 million per patient, corresponding to 1 to 2 million per kilogram of body weight ^55^. This may in part explain the huge discrepancy of outcome between preclinical and clinical studies, assuming that the therapeutic benefit is dose-dependent ^21^. Although the notion of increasing MSC dose might be tempting, safety should be assessed carefully for it might, for example, increase the risk for embolism or adverse reactions. In addition, the lack of standardized pharmacodynamics and pharmacokinetics models applied to MSCs represents a limiting factor ^56^.

Another potential explanation for the translational gap between clinical and preclinical data is that, in patients, the degree of severity might be too high for MSC therapy to be as efficacious as in animal studies. In order to get better clinical outcomes, MSC-based therapy may be considered as prevention treatment together with first-line therapy and not only as salvage or even palliative therapy. However, this notion will require proper risk–benefit evaluation and support from ethics committees.

### Hemocompatibility and immune compatibility

For a long time, MSCs have been considered to be immune-privileged, allowing their transplantation across histocompatibility barriers ^57^. Recent data, however, indicate that MSC transplantation may provoke donors’ humoral and cellular immune responses, especially in allogeneic settings ^21, 58^. In GvHD, in fact, this immune recognition appears to be fundamental for the therapeutic effect: MSCs recognized by cytotoxic T cells undergo apoptosis and are phagocytosed by macrophages which subsequently elicit immunosuppression via prostaglandin E _2_ (PGE _2_) and IDO activities ^59– 61^.

MSCs have also been shown to elicit an instant blood-mediated inflammatory reaction (IBMIR) in both a cell dose- and a donor-dependent manner ^62^. Non-bone marrow-derived MSCs appear to express higher levels of pro-coagulant tissue factor, which makes them more likely to induce IMBIR ^63^. Adding anti-coagulants during MSC transplantation may be a good option for clinical application ^36, 52^. Likewise, the selection of tissue factor–negative or low expressing MSCs has been proposed as a strategy to improve hemocompatibility ^63^.

Moreover, allogeneic MSC transplantation can provoke an adaptive immune response in mice through increased T-cell memory and allo-antibodies ^64, 65^. The latter may be associated with complement-mediated cytotoxicity ^58^. Yet only two clinical trials ^1, 67^ report the development of donor-specific antibodies in patients who have received allogeneic MSCs ^66^. But the authors argue that the increased allo-antibodies have no relevance in the clinical outcome or the occurrence of adverse events ^1, 67^. Moreover, in two other clinical trials, which used allogeneic MSCs to treat type 2 diabetes and diabetic nephropathy, there was no report of patients developing donor-specific HLA antibodies despite donor–recipient mismatching ^68, 69^. Although the results from clinical studies seem encouraging, the scarcity of studies elucidating possible allo-immune reactions may cause a bias in the observed trend ^66^. The occurrence of FBS (used as culture supplement for cell expansion)-specific antibodies has prompted the search for alternative and improved culture conditions as described above ^70^.

## Mechanisms of action

### Mesenchymal stromal cell secretome

**Figure 2.**
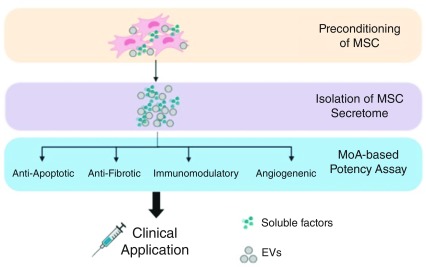
Mesenchymal stromal cell (MSC) secretome in clinical application. Strategies to harness the MSC secretome for clinical purposes include preconditioning/priming of MSCs to manipulate their paracrine factors, isolation of the secretome, and establishing MoA-based potency assay. EV, extracellular vesicle; MoA, mechanism of action.

The MSC secretome is composed of different soluble factors, including cytokines, growth factors, chemokines, immunomodulatory molecules, cell organelles, and nucleic acids, which are produced, some of these eventually encapsulated in extracellular vesicles, and secreted or directly transferred to neighboring cells ^71, 72^. These factors can modulate the immune system, inhibit cell death and fibrosis, stimulate vascularization, and promote tissue remodeling ( Figure 2) ^73^. MSCs can adapt efficiently to the local milieu and change their secretome ^74^. On one hand, this significantly hampers the understanding of their mechanisms of action (MoAs) *in vivo* and the establishment of predictive and quantitative potency assays. On the other hand, it paves the way to potentially improve therapeutic efficacy (for example, the preconditioning with different factors that activate very specific signaling pathways). Treating MSCs *in vitro* with hypoxia, 3D culture, or soluble factors such as stromal cell–derived factor 1 (SDF-1) or transforming growth factor-beta (TGF-β) triggers Akt, ERK, and p38MAPK signaling pathways. These pathways, at the same time, can induce the production of cytoprotective molecules (catalase, heme oxygenase-1, and so on), pro-regenerative (basic fibroblast growth factor, hepatocyte growth factor, insulin-like growth factor-1, and so on) and pro-angiogenic (vascular endothelial growth factor, or VEGF) factors, and also immunomodulatory cytokines (IDO, PGE _2_, interleukin-6, and so on) ^42, 71^.

### Extracellular vesicles

Extracellular vesicles (EVs) are further candidates to explain the therapeutic effects of MSCs. EVs are membrane-enclosed particles of different sizes (exosomes, microvesicles, and apoptotic bodies) released by cells in the plasma and other body fluids ^75, 76^. EVs transport biologically active molecules and genetic information to target cells, influencing their function ^77^. Thanks to these characteristics, EVs are also emerging as biomarkers for various diseases ^78^. EVs carry a wide variety of genetic material, in particular microRNAs, which play an important role in the biological function of EVs. These small RNAs regulate the cell cycle and migration (for example, miR-191, miR-222, miR-21, and let-7a), inflammation (for example, miR-204-5p), and angiogenesis (for example, miR-222 and miR-21). In a new therapeutic approach, MSC-derived EVs are being engineered by increasing or modifying their content (proteins or RNA) ^77^. As an example, an effective drug delivery system for wound healing in diabetes was developed by transfecting non-coding RNA (Lnc-RNA-H19) into EVs ^79^. Based on these data, some researchers suggest that the conditioned medium or even EVs should be used as drugs rather than MSCs ^80^.

In some circumstances (for example, GvHD mentioned above), dead or dying cells may contribute to therapeutic efficacy. Thus, an improved understanding on MSC “necrobiology” has been proposed, considering apoptosis, autophagy, mitochondrial transfer, and also vesicles ^61^. Recognition by the innate immune system in different disease contexts may be key to understand and improve MSC function ^60, 81^.

### Potency assays

For advanced clinical trials, assays that can verify MSC identity and quality and can predict their functionality *in vivo* are required ^82^. Owing to the manifold functions of MSCs and their rapid adaptation to the local milieu, which may modify their function at sites of injury, disease, or inflammation, assays to predict these functions *in vivo* are hard to develop.

Agreed quality-control criteria include the determination of presence and absence of certain surface markers and of MSC differentiation potential, their senescence status, their secretome and immunomodulatory functions. In addition, surrogate assays which more specifically test the proposed therapeutic mechanism of action, for example angiogenesis have been established ^83– 87^. The group of Galipeau was the first to suggest a combinatorial assay matrix as a platform to integrate different assays ^88, 89^. In the first study, they employed secretome analysis and quantitative RNA-based array to estimate the immunomodulatory capacity of MSCs and their crosstalk with peripheral blood mononuclear cells (PBMCs), in which CXCL9, CXCL10, VEGF, and CCL2 secretion and expression were correlated with suppression of T-cell proliferation ^88^. The other study investigated the phosphorylation of signal transducer and activator of transcription (STAT) in MSC-PBMC co-culture settings where STAT1 and STAT3 phosphorylation was associated with MSC immunoinhibitory capacity ^89^. Moreover, Phinney *et al*. reported a “Clinical Indications Prediction Scale” that, based on Twist-1 expression levels, could predict therapeutic efficacy: high levels of Twist-1 predict higher angiogenic potential, whereas low levels are in line with improved anti-inflammatory and immunosuppressive actions ^90^.

We expect rapid progress in the development of combinatorial potency assays based on the increasing knowledge of MSC biology (omics, including single-cell analyses) ^91, 92^. Integrating this more comprehensive insight into MSC heterogeneity with MSC molecular signatures and their highly complex interaction with the local microenvironment in line with a better understanding of molecular mechanisms of action in various pathological settings will hopefully enable easy-to-perform assays with predictive value.

## Future perspectives

The key questions for the future may be, do we need cells? Do we need viable cells, or do apoptotic cells and subcellular components such as EVs, or mitochondria, or just the secretome do a similar job?

The biological properties of the MSC secretome and how it orchestrates MSC immunomodulatory and regenerative capacity in the disease context remain enigmatic, prompting further studies. Moreover, given the possibility of modulating MSCs and their secretome, a disease-specific MSC priming (for instance, with pro-inflammatory cytokines) may improve efficacy.

Lastly, in order to standardize MSC therapy and avoid outcome bias, rigorous potency assays are needed. However, the selection of specific potency assays, whether it is a disease-specific (for example, angiogenesis and immunomodulation) or a more general (for example, proliferation) evaluation of MSC function that can be used regardless of diseases’ pathophysiology, needs further elucidation.

Given the enormous knowledge gain in MSCs over the past years, largely obtained by bench-to-bedside-and-back approaches and recapitulated by the continuous adaptation of the term MSCs (“name follows function”), we expect the development of novel translational strategies. A better understanding of failures, the identification and consequent mitigation of challenges and finally an improved understanding of MoAs, translating into robust potency assays, will be key for a successful translation of MSCs into clinical practice.
